# 

**Published:** 1888-08

**Authors:** 


					﻿Vol. IX.
AUGUST, 1888.
No. 8.
TH HZ
iwmiiimiPratiiiiiW:
AX /Vi OiNTffT.Y xl OTTK W TL.
DEVOTED TO
DENTAL AND ORAL SCIENCE.
W. XAVIER SUDDUTH, M. D., D. D. S,
EDITOR.
The International Dental Journal Association,
PUBLISHERS,
51 West 37th Street, New York City,
AND
208 Franklin Street, Buffalo, N. Y.
$2.50 Per Annum in Advance, .... Single Copies, 25 Cents.
Entered at the Post-Office, Buffalo, N. Y., as Second-Class matter.
				

